# Applying Hot Compresses with Rhubarb and Mirabilite to Reduce Pancreatic Leakage Occurrence in the Treatment of Severe Acute Pancreatitis

**Published:** 2017-01

**Authors:** Yunxia WANG, Xinhui ZHANG, Chunying LI

**Affiliations:** 1. Dept. of Hepatobiliary Surgery, Liaocheng People’s Hospital, Shandong, 252000, China; 2. Dept. of Hemopathology, Liaocheng People’s Hospital, Shandong, 252000, China

## Dear Editor-in-Chief

According to traditional Chinese medicine theory (1), pancreatitis is a “splenic precordial pain” or “hypochondriac pain,” caused by excessive drinking, gluttony, or gloomy mood, or secondary to biliary tract ascariasis, gallstones, and other diseases. To date, no specific drugs have been able to cure acute pancreatitis, and management is typically conservative. Traditional Chinese and Western medicine, when used in combination, can achieve good results.

We explored the effectiveness of nursing with the application of hot compresses of rhubarb and mirabilite in reducing the occurrence of pancreatic leakage in the treatment of severe acute pancreatitis. We included 96 patients with severe acute pancreatitis diagnosed at our hospital from September 2013 to September 2015, and randomly divided them into a control group (n = 47) and an intervention group (n = 49). The control group received standard nursing care and therapy, while the intervention group received a combination of rhubarb infusion and hot compresses with rhubarb and mirabilite. Rhubarb is cold in nature; it prevents blood stasis, promotes heat removal and purgation, and breaks stagnation. These effects are pivotal for eliminating excess heat, enhancing indigestion, and stimulating metabolism (2, 3). Rhubarb can clear the intestinal tract, lower intra-abdominal pressure, reduce endotoxin-induced intestinal mucosa injuries, reduce the secretion of pancreatic amylase, eliminate inflammatory edema, and reduce IL-6 levels in the blood (4, 5). In contrast, mirabilite is salty, bitter, and cold in nature, usually processed into crystals. Because patients with pancreatitis gather heat in the *Fu*, which can obstruct of *Fu Qi*, using external hot compress can eliminate heat, toxins, stagnation, and edema from extravasated blood. Moreover, it can quickly relieve the patients’ clinical symptoms of abdominal pain and distension (6–8).

As mentioned, patients in the intervention group were treated with traditional Chinese medicine internally and externally. First, 20 g of rhubarb was soaked in 200 ml of boiled water for 30 min until the water turned dark brown. The raw rhubarb was then filtered out and the remaining water was cooled down to a temperature appropriate for ingestion. Subsequently, 100 ml of the medicinal liquid was given to the patient through an indwelling gastric tube, which was then closed for 1 h. This 100 ml dose was repeated two times a day for a week. An additional 100 ml of the prepared medicinal liquid was given as retention enema. A lubricant was used to facilitate the insertion of 25–30 cm of a one-time anal tube and the liquid was injected using a syringe. Subsequently, the tube was removed, and patient was asked to lie on their back for 30 min.

External treatment involved hot compressed with rhubarb and mirabilite. A cotton compress was made into an elongated bag with a zipper in the upper mouth and with wide strips sewn in the middle. Next, 500–1000 g of mirabilite was grounded into fine foam and fried until hot. This mixture was then placed into the bag and applied to the patient’s abdomen. We paid close attention to the temperature in order to avoid skin burns. The abdominal skin was examined every other hour, and the bag was replaced every 24 h. This procedure was repeated until the patient reported a relief in the abdominal pain and distension. SPSS v19.0 (IBM Corp., Armonk, NY, USA) was used for data analyses. Measurement data were expressed as mean ± standard deviation. For comparison between groups we used independent sample t-tests, while that within the groups was by paired t-tests. Enumeration data are expressed by rate, and comparison between the groups was tested by χ^2^ tests, while ranked data were tested by ranked-sum tests. *P* < 0.05 indicated statistical significance.

The times taken for abdominal distension, abdominal pain, and blood and urinary amylase values to normalize were significantly shorter in the intervention group compared with those in the control group. By the end of the observation period, the white blood cell count, C-reactive protein level, and interleukin-6 level were reduced in both groups; however, the decline was more obvious in the intervention group (Table 1). In addition, the overall and full absorption efficiency rates were significantly higher in the intervention control group. Nursing satisfaction scores, as well as the rate and degree of satisfaction in the intervention group, were all higher than those in the control group (Table 2). The differences were statistically significant (*P* < 0.05).

**Table 1:** Comparison of serum inflammatory indicators

** Group**	** WBC count (109/L)**		** CRP (mg/L)**		** IL-6 (μg/L)**	
	**Before intervention**	**After intervention**	**Before intervention**	**After intervention**	**Before intervention**	**After intervention**
Intervention	16.5 ± 2.2	6.6 ± 1.3	13.4 ± 2.6	3.8 ± 1.5	65.9 ± 12.5	21.5 ± 8.3
Control	16.3 ± 2.3	10.5 ± 2.0	13.2 ± 2.7	8.6 ± 1.6	62.3 ± 14.2	34.6 ± 7.6
* t *	0.242	5.326	0.421	5.766	0.632	5.447
* P *	0.637	0.023	0.567	0.018	0.527	0.021

Data shown as mean ± standard deviation

Abbreviations: CRP, C-reactive protein; IL-6, interleukin-6; WBC, white blood cell

**Table 2:** Comparison of satisfaction with nursing care

**Group**	**Cases**	**Satisfaction degree score**	**Very satisfied**	**Satisfied**	**Unsatisfied**	**Satisfaction rate**
Intervention	49	8.6 ± 1.7	20 (40.8)	22 (44.9)	7 (14.3)	42 (85.7)
Control	47	6.4 ± 1.3	13 (27.7)	19 (40.4)	15 (31.9)	32 (68.1)
* t *		6.245		6.203		4.221
* P *		0.010		0.045		0.040

Data shown as cases (%) or as mean ± standard deviation

In conclusion, applying hot compresses with rhubarb and mirabilite to nurse patients with severe acute pancreatitis can promote the absorption of pancreatic exudates and shorten the recovery time of patients, implying that this method is valuable to clinical practice.
